# Spatiotemporal Analysis of Land Use and Cover Dynamics in Protected Areas of the Brazilian Cerrado

**DOI:** 10.1155/sci5/7984448

**Published:** 2025-04-25

**Authors:** Mariele R. Torgeski, Emerson M. de Carvalho, Alex M. dos Santos, Ana P. Lemke, Claudemir A. G. Fioratti, Mauricio Stefanes, Sandro M. Silva, Rosilda M. Mussury

**Affiliations:** ^1^Postgraduate Program in Biodiversity and Environmental Science, Faculty of Biological and Environmental Sciences, Federal University of Grande Dourados, Dourados, Mato Grosso do Sul, Brazil; ^2^Center of Agroforestry Sciences and Technologies (CFC-TA), Federal University of Southern Bahia, Itabuna, Bahia, Brazil; ^3^Faculty of Biological and Environmental Sciences, Federal University of Grande Dourados, Dourados, Mato Grosso do Sul, Brazil

## Abstract

The Brazilian Cerrado, renowned as the most biodiverse savanna, is characterized by its pronounced climatic seasonality, diverse vegetation mosaic, and distinct topographic variations. Natural protected areas (NPA) within this biome play a pivotal role in safeguarding both biodiversity and natural resources. The objective of this study is to analyze the dynamic shifts in land use and land cover across seven NPA and their corresponding buffer zones (BZs) within a Cerrado region situated in Central-West Brazil. This research encompasses a comprehensive multitemporal analysis of satellite imagery spanning the period from 1985 to 2018 utilizing a geographic information system (GIS). The variability in land use and land cover classes is considerably more constrained within the NPA than BZs. The Templo dos Pilares Municipal Natural Park exhibits substantial expanses of preserved vegetation, while the Nascentes do Rio Taquari State Park demonstrates an extensive prevalence of pasturelands. The NPA exhibit coherent patterns of land cover transformation within their respective BZs. However, alterations in the landscape within the BZs offer insights into potential forthcoming challenges to the NPA. Escalated land use within the surrounding matrix of protected areas presents a formidable obstacle to biodiversity conservation, owing to extraneous pressures. A comprehensive understanding of the spatiotemporal distribution of land use and land cover within safeguarded Cerrado regions contributes substantively to the augmentation of management, preservation, and conservation endeavors over time.

## 1. Introduction

Transformations in landscapes on a global scale bring to light the debate about the impacts of human activities on natural resources, especially changes in land use/cover (LULC) that strongly affect river basins [1], in addition to impacts on surface–atmosphere interactions [2], on traditional communities [3], on terrestrial biomes [4], and on water availability [5], among others. For example, the scarcity of water for family farming in West Africa led to a change in land cultivation [5] and in the Brazilian Amazon, pastureareas for cattle ranchingexpanded by 46% between 1985 and 2017, while agricultural land increased by 172% over the same period [4]. These transformations cover several areas, from physical aspects, such as topography and vegetation cover, to socioeconomic aspects, such as urbanization and worsening climate change.

Brazil has experienced changes in land cover over the last 34 years, affecting surface–atmosphere interactions in most biomes [2]. Therefore, changes in land use and coverage in Brazil are a topic of great relevance due to the country's territorial extension and the diversity of ecosystems present. Therefore, such dynamics, in Brazil, have significant consequences for the environment, economy, and society.

The Cerrado, the second-largest biome in Latin America, encompasses an expansive territory of approximately 2 million square kilometers. In terms of size, it yields only to the Amazon rainforest. Geographically situated at the heart of the continent, the Cerrado serves as a connective link between the Chaco to the southwest and the Caatinga to the northeast. This region function as a natural corridor that separates the two most salient American tropical forest domains: the Amazon rainforest to the northwest and the Atlantic Forest to the southeast [6]. Recognized as one of the globe's biodiversity hotspots, the Cerrado harbors significant species richness and endemism, despite its susceptibility to anthropogenic threats [7], with certain local areas showing increasing deforestation [8].

The primary strategy to counteract the encroachment of human habitation into the Cerrado involves the design, establishment, and governance of protected natural areas. Within this framework, Natural Protected Areas (NPA) stand out as the most efficacious mechanism for safeguarding biodiversity [9]. NPA, as legally designated spaces, are instituted with the explicit objective of ensuring biodiversity protection and manifest in two distinct forms: Integral Protection and Sustainable Use [10].

The NPAdatabase maintained by the Brazilian Ministry of Environment and Climate Change point out to Cerrado encompasses a total of 481 NPA, including federal, state, and municipal areas, those area safeguarding approximately 9% of the biome's extension and approximately 18% of the cumulative NPA throughout Brazil. Notably, a mere 32% of those areas within the Brazilian Cerrado area classifiedas Integral Protection [11], in stark contrast to 1.6% of the territory within the state of Mato Grosso do Sul [12].

Numerous NPA experience the repercussions of their proximity to external factors, resulting in disruptions to ecological processes that extend to within the confines of the protected area [13–15]. To mitigate the potential impacts of this proximate relationship, legislation mandates the shielding of the environs encompassing NPA, known as the buffer zone (BZ), as defined by Article 2, Clause XVIII of the Brazilian National System of Conservation Units (Federal Law No. 9.985/2000). The BZ is delineated as the “surroundings of a conservation unit where human activities are subject to specific rules and restrictions with the purpose of minimizing negative impacts on the unit.” This buffer serves as a protective shield to counteract the adverse effects emanating from adjacent domains, such as the propagation of environmental fragmentation, invasive exotic species, and edge effects to the interior of NPA [16].

Environmental studies, particularly within NPA and their peripheries, constitute essential endeavors contributing to the formulation of strategies and public policies aimed at the establishment, governance, management, and conservation of these domains. The mere designation of a NPA, however, does not suffice to guarantee its effective role in biodiversity conservation [17]. To monitor alterations within these regions, spatial data provide a mechanism to assess transformations in land use and land cover through digital processing of remotely sensed imagery [18]. The discernment of these patterns provides insights for the evaluation of anthropogenic shifts and documentation of land use trends over time [19].

As indicated by the Interactive System for Environmental Licensing Support (SISLA), the original extent of the Cerrado in Mato Grosso do Sul exceeded two million square kilometers, encapsulating approximately 24% of the state's terrain. However, between 2001 and 2017 alone, it sustained a depletion of just over 19,000 square kilometers [20]. Data published by the National Institute for Space Research (INPE) underscore that between August 2020 and July 2021, a substantial expanse amounting to nearly 288 square kilometers was lost. Given these statistics, a comprehensive understanding of land use and land cover patterns within NPA, their BZs, and the surrounding land use matrix is a prerequisite for fostering the effective management of these domains.

Recent research results have pointed to the fact that deforestation in the Cerrado has reached alarming rates in recent years [21]. Despite projections of a severe extinction event, a window of opportunity is now open for a combination of policies to prevent biodiversity collapse in the Cerrado hotspot [22]. Given this, could NPAbe contributing to the maintenance of the Cerrado landscape and, consequently, to the maintenance of its biodiversity? To answer this question, our hypothesis is based on the idea that CUs of the Cerrado Biome located in the central portion of Brazil can contribute to mitigating the process of transformation of Cerrado landscapes.

## 2. Materials and Methods

### 2.1. Study Area

The study area is located in the central portion of Brazil, in the Cerrado Biome. According to Strassburg et al. [22], this biome lost around 46% of its native vegetation area. This process intensified from 1970s onwards, when public policies began to promote the expansion of the agricultural frontier and the modernization of farming activities [21]. Thus, an area in the southern portion of the Cerrado Biome, states of Goiás and Mato Grosso do Sul, was selected for analysis.

**Figure 1:**
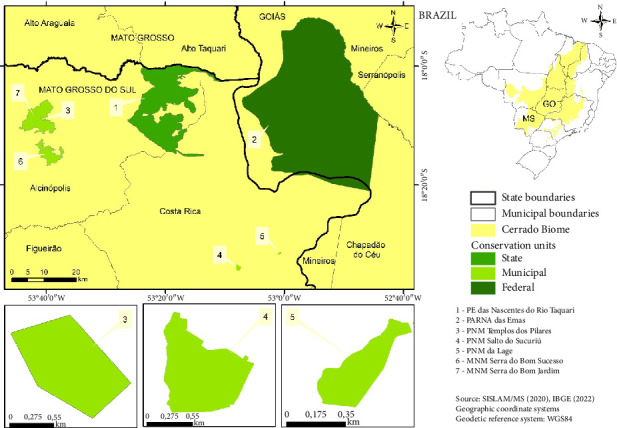
Geographical depiction of the study area and the encompassed conservation units.

The study area encompasses NPA situated in northern Mato Grosso do Sul, along its border with the state of Goiás (Figure 1).

The largest among these units is Emas National Park (ENP), predominantly situated in the state of Goiás, covering a vast expanse of 133,069 hectares. The remaining NPA are exclusively situated within the state of Mato Grosso do Sul (Table 1).

The study area exhibits a distinctive landscape characterized by monocultures of cotton, soybean, corn, and other grains intermingled with diverse Cerrado vegetation. These include open-canopy formations, such as grassland and rocky grassland, and closed-canopy formations, such as cerrado woodland and deciduous seasonal forest.

## 3. Methods

Vector files corresponding to the boundaries of the NPA in shape file format, alongside their respective BZs (BZs), were procured from the digital repositories of the Interactive System for Environmental Licensing Support (SISLA). These records are accessible on the website of the Mato Grosso do Sul Institute of Environment Management [23], as well as through the National Classification of NPAs, retrievable via the URL https://mapas.mma.gov.br/i3geo/datadownload.htm. For the Serra do Bom Sucesso Municipal Natural Monument (MNMSBS), which does not have a management plan and consequently lacks BZ information, adherence to the prescribed BZ of 3 km was observed, in accordance with CONAMA Resolution No. 428 dated 12/17/2010. To delineate the land use matrix, a buffer of 10 km was established from the perimeters of the BZ of each NPA.

The elucidation of changes in land use and land cover entailed the consideration of historical series for mapping. These image series comprise 26 scenes from the Landsatsatellite missions, corresponding to path/row, 225/072, 225/073, 226/072 and 226/073, obtained from the United States Geological Survey (http://glovis.usgs.gov/). Each scene includes bands 4 (Blue), 5 (Green) and 6 (Red), which were combined into false-color composites with a spatial resolution of 30 meters (https://glovis.usgs.gov/). The dataset spans from ten years preceding the establishment of the NPÁ up to 2018. The selection of satellite images was contingent on the dry season (May to September) to circumvent cloud interference and ensure optimal differentiation between vegetation structures, thus minimizing seasonal perturbations.

The digital processing of images adhered to the methodology proposed by Nunes et al. [25]. The sequential stages encompassed database creation, image procurement, colors composition, mosaic formation, clipping of the areas of interest, classification, and performance analysis. All procedures in this phase, including the development of the land use and land cover maps, were carried out using the ArcGis 10.5 software [24].

The establishment of the geographic database was tied to the WGS84 datum. Band composition entailed the utilization of the red (R), green (G), and blue (B) systems, executed through false color representation. For Landsat generations 5 and 7, bands 3, 4, and 5 were used, whereas Landsat generation 8 incorporated bands 5, 6, and 4. Mosaic construction involved the amalgamation of two scenes for each year. Clipping procedures were executed to delineate the specific areas of the NPA and BZs. The supervised classification approach was adopted for image processing, employing the maximum likelihood (MAXVER) algorithm. This was followed by an assessment of the performance and accuracy of the classification results.

During the image classification phase, six discrete classes pertaining to land use and land cover were defined. These classes encompass Forest, Savanna, Pasture, Temporary crop, Urban infrastructure, and water. Subsequent to classification, performance was evaluated using the kappa coefficient. The kappa coefficient values were classified based on thresholds delineated by Landis and Koch [26], as detailed below:

• Kappa < 0: no agreement
• 0% to 20%: minimal agreement
• 21% to 40%: reasonable agreement
• 41% to 60%: moderate agreement
• 61% to 80%: substantial agreement
• 81% to 100%: perfect agreement

For individual class analysis, user's accuracy statistics were employed. This metric was determined as the ratio of accurately classified samples for each class to the total number of samples within that class.

A total of 185 control points were meticulously documented in the study area using a Global Positioning System (GPS) satellite navigator to ensure precision in field data acquisition. The primary objective was to characterize the local landscape and establish an accurate correlation between the thematic classes defined through image interpretation and the actual vegetation cover patterns observed in situ.

The selection of data collection points was based on the need to ensure the spatial representativity of different land use and land cover classes, as well as to validate the supervised classification applied to remote sensing images. The acquisition of primary data through field visits enabled the direct verification of information extracted from digital image processing, contributing to a more precise categorization of the landscape and minimizing potential classification errors.

The points were georeferenced using GPS and recorded in electronic spreadsheets, facilitating a detailed comparative analysis with the results obtained in the laboratory. This methodological approach, widely adopted in environmental studies, allowed for the triangulation of data from multiple sources, enhancing the reliability of spatial inferences and improving the understanding of landscape transformation dynamics.

Thus, the integration of remote analysis and field validation strengthened the robustness of the research, ensuring that variations in land use and land cover were detected with greater accuracy and solid technical–scientific support.

## 4. Results

The results from the error matrix enabled the identification of a kappa coefficient of 77.2%. This coefficient corresponds to a substantial level of agreement, where the classes with the highest degree of pixel confusion were Pasture (user's accuracy of 71.4%) and Grassland (58.8%), as presented in Table 2.

**Figure 2:**
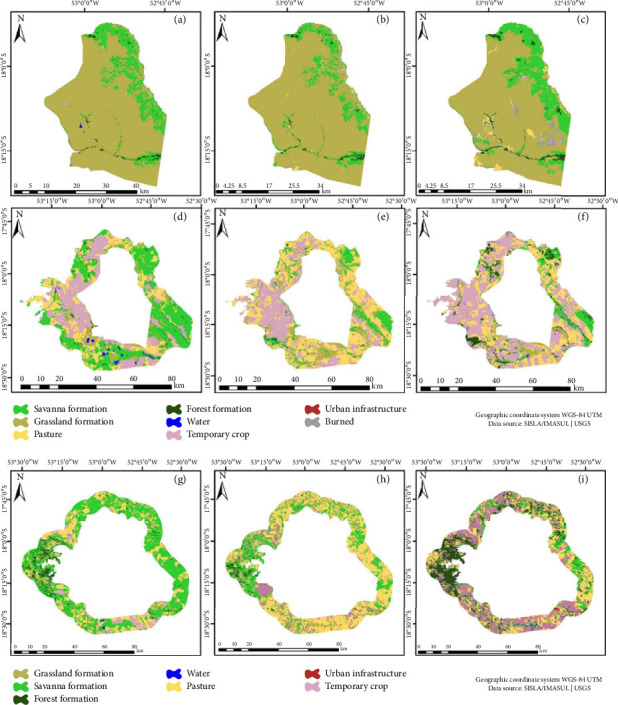
Emas National Park (ENP). Land use and cover map: (a) 1985, (b) 1995, and (c) 2018. Buffer zone: (d) 1985, (e) 1995, and (f) 2018. Land use matrix: (g) 1985, (h) 1995, and (i) 2018.

Regarding ENP, a noteworthy expansion in the Savanna class of 2.93% was found. Additionally, Pasture areas proliferated across the southern and extreme northern sectors (Figures 2(a), 2(b), and 2(c)). Furthermore, the last year of analysis witnessed a surge in the burned class. In the vicinity of the BZ, a noticeable decline in natural vegetation classes was noted, accompanied by an approximately 18.78% increase in anthropogenic classes (pasture and temporary crop) (Figures 2(d), 2(e), and 2(f)). Notably, the land use matrix revealed an expansion of the Temporary crop class area from 17,961 hectares in 1985 to 105,349 hectares in 2018 (Figures 2(g), 2(h), and 2(i)).

**Figure 3:**
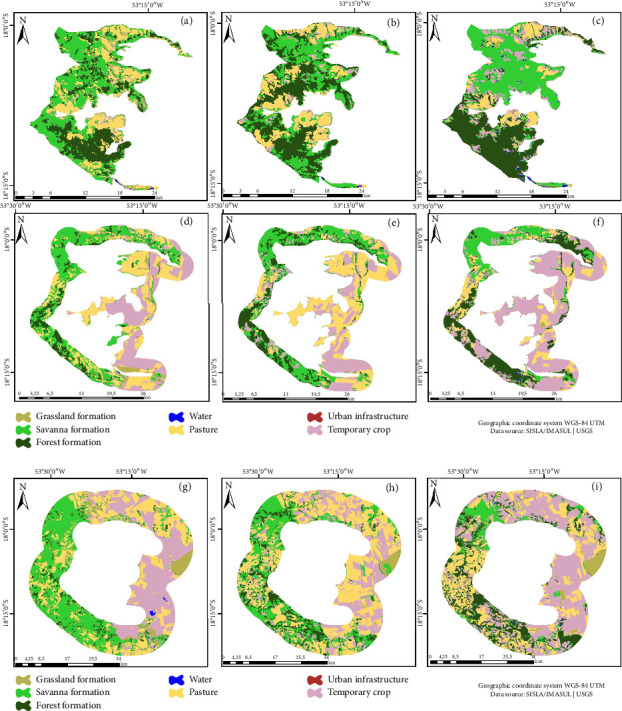
Nascentes do Rio Taquari State Park (PENRT). Land use and cover map: (a) 1989, (b) 1999, and (c) 2018. Buffer zone: (d) 1989, (e) 1999, and (f) 2018. Land use matrix: (g) 1989, (h) 1999, and (i) 2018.

Within the Nascentes do Rio Taquari State Park (PENRT), an increase in the forest class by 11,609 hectares during the period from 1989 to 2018 was observed (Figures 3(a), 3(b), and 3(c)). Conversely, a reduction of 11,043 hectares was recorded for the Savanna class. The year 1999, marking PENRT's inception, witnessed an increase in the forest class, trailed by Savanna and Pasture. In 1989, within the BZ (Figures 3(d), 3(e), and 3(f)), Pasture (31.06%), Savanna (25.95%), and Temporary crop (27.15%) were the predominant classes. Examining the land use matrix of the PENRT, Temporary crop (30.07%), Savanna (28.66%), and Pasture (27.29%) stood out (Figures 3(g), 3(h), and 3(i)).

**Figure 4:**
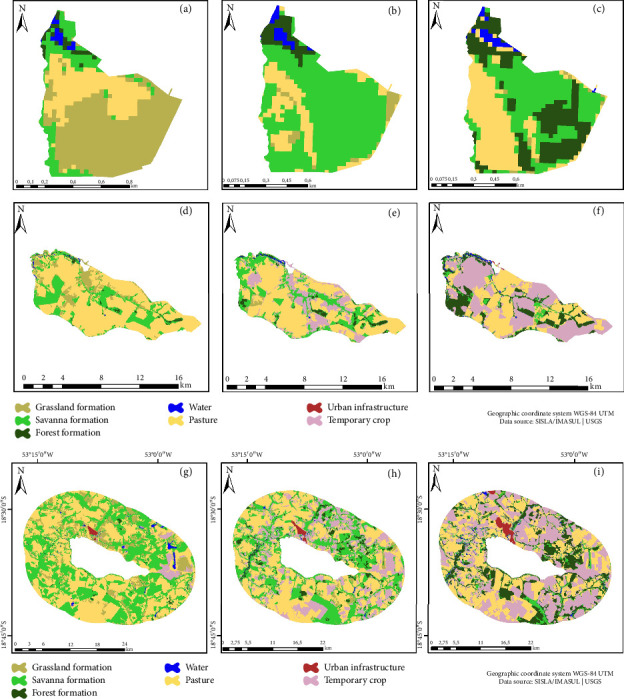
Salto do Sucuriú Municipal Natural Park (PNMSS). Land use and cover map: (a) 1990, (b) 2000, and (c) 2018. Buffer zone: (d) 1990, (e) 2000, and (f) 2018. Land use matrix: (g) 1990, (h) 2000, and (i) 2018.

For the Salto do Sucuriú Municipal Natural Park (PNMSS), the Savanna class emerged as the dominant class (65.11%), followed by Pasture (18.63%), as shown in Figures 4(a), 4(b), and 4(c). Within the park's BZ, Pasture (50.76%), Savanna (21.54%), and Temporary crop (20.37%) were evident. Analyzing the land use matrix revealed a contraction of the Savanna class (28.67%) and a commensurate expansion in the Temporary crop class (34.68%) (Figures 4(g), 4(h), and 4(i)).

**Figure 5:**
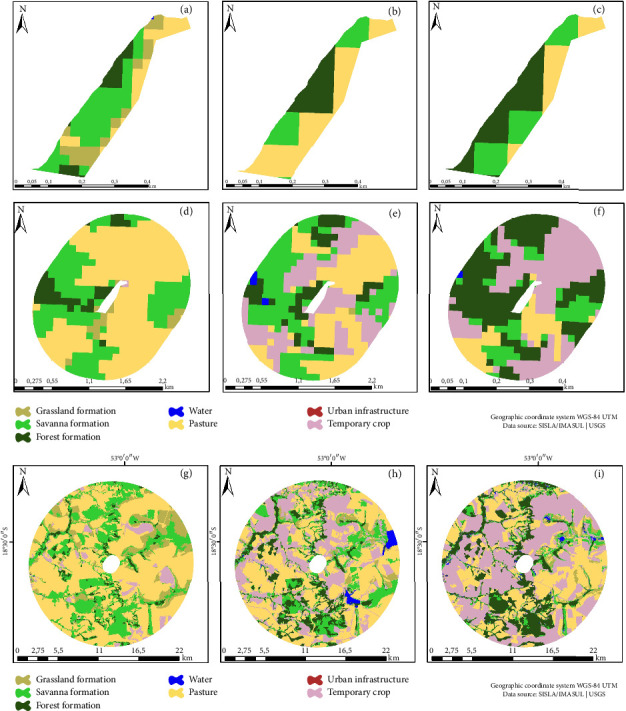
Salto da Lage Municipal Natural Park (PNML). Land use and cover map: (a) 1991, (b) 2001, and (c) 2018. Buffer zone: (d) 1991, (e) 2001, and (f) 2018. Land use matrix: (g) 1991, (h) 2001, and (i) 2018.

Salto da Lage Municipal Natural Park (PNML), characterized by its modest area, experienced a 21.13% increase in the forest class from 1991 to 2018. Additionally, the pasture class increased by 11.27% (Figures 5(a), 5(b), and 5(c)). In the BZ (Figures 5(d), 5(e), and 5(f)), the Temporary crop class expanded by 35.85%, while the Savanna class contracted by 18.21%. The land use matrix reflected a comparable trend, depicting an increase in the area of the Temporary crop class of 31.43% from 1991 to 2018, coupled with a decline of 9.03% in the Savanna class (Figures 5(g), 5(h), and 5(i)).

**Figure 6:**
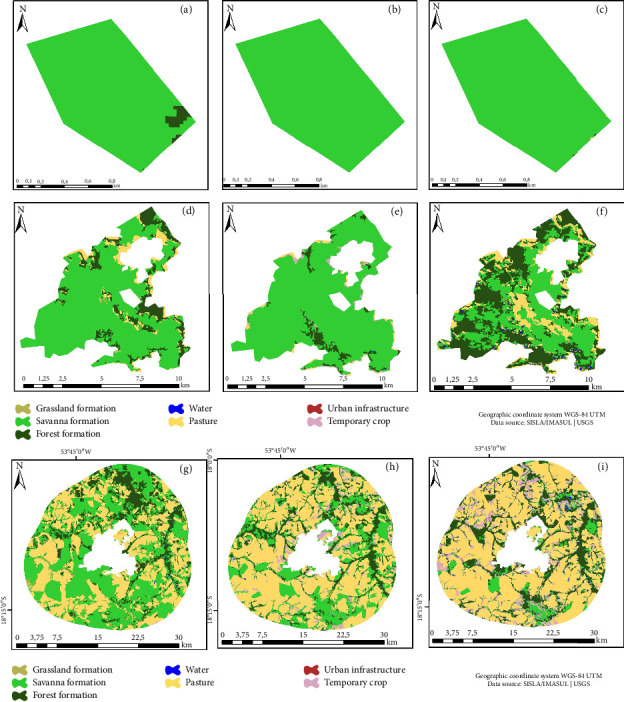
Templo dos Pilares Municipal Natural Park (PNMTP). Land use and cover map: (a) 1993, (b) 2003, and (c) 2018. Buffer zone: (d) 1993, (e) 2003, and (f) 2018. Land use matrix: (g) 1993, (h) 2003, and (i) 2018.

The Templo dos Pilares Municipal Natural Park (PNMTP), initially characterized by a Savanna land cover of 89.61% in 1993, experienced a slight decrease to 88.74% by 2018 (Figures 6(a), 6(b), and 6(c)). In the BZ, there was a noteworthy increase in the Forest class by 18.23% between 1985 and 2018 (Figures 6(d), 6(e), and 6(f)). In the context of the land use matrix, an expansion of the Pasture class by 14% from 1985 to 2018 was evident (Figures 6(g), 6(h), and 6(i)).

**Figure 7:**
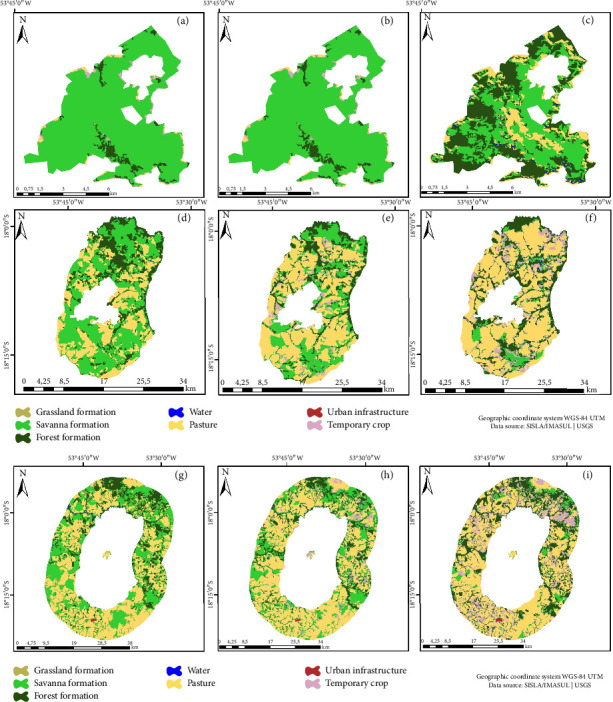
Serra do Bom Jardim Municipal Natural Monument (MNMSBJ). Land use and cover map: (a) 1993, (b) 2003, and (c) 2018. Buffer zone: (d) 1993, (e) 2003, and (f) 2018. Land use matrix: (g) 1993, (h) 2003, and (i) 2018.

The Serra do Bom Jardim Municipal Natural Monument (MNMSBJ) experienced a reduction in the Savanna (28.68%) and Pasture (5.16%) classes from 1993 to 2018 (Figures 7(a), 7(b), 7(c), 7(d), 7(e), and 7(f)). Notably, the period from 2003 onward witnessed substantial increases in the Temporary crop and Pasture classes within the BZ and land use matrix (Figures 7(g), 7(h), and 7(i)).

**Figure 8:**
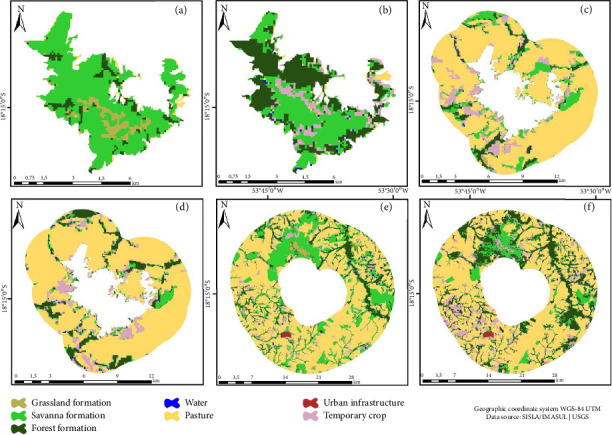
Serra do Bom Sucesso Municipal Natural Monument (MNMSBS). Land use and cover map: (a) 2008; (b) 2018. Buffer zone: (c) 2008; (d) 2018. Land use matrix: (e) 2008; (f) 2018.

Comparatively, the Serra do Bom Sucesso Municipal Natural Monument (MNMSBS), exhibited relatively well-preserved natural vegetation when juxtaposed with its BZ (Figures 8(a), 8(b), 8(c), and 8(d)). The dynamic transformation of the landscape materialized as changes across specific classes: an expansion of 25.98% in the Forest class, a marginal reduction of 1.31% in the Pasture class, and a 6.6% increase in the Grassland class. This trend extended to the land use matrix, revealing a decrease of 11.46% in the Savanna class and a simultaneous increase of 4.45% in the Temporary crop class by 2018 (Figures 8(e) and 8(f)).

**Figure 9:**
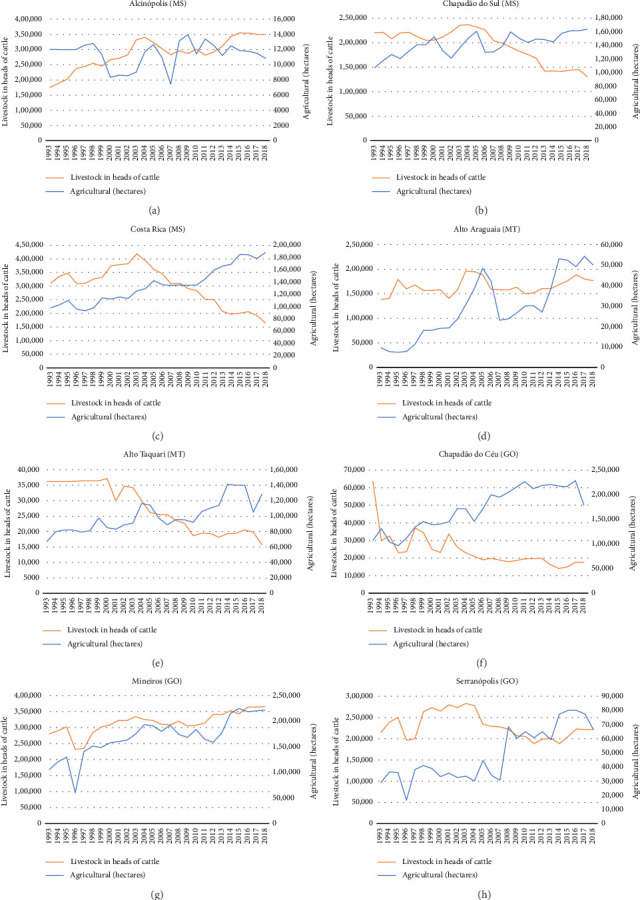
Evolution of municipal agricultural production (in hectares) and cattle livestock (in number of heads) from 1993 to 2018. Available data on cultivated area and livestock are only available from 1993 onwards for all municipalities analyzed. The figure presents the following municipalities: (a) Alcinópolis (MS), (b) Chapadão do Sul (MS), (c) Costa Rica (MS), (d) Alto Araguaia (MT), (e) Alto Taquari (MT), (f) Chapadão do Céu (GO), (g) Mineiros (GO) and (h) Serranópolis (GO). Source: IBGE, 2018.

Figures 9(a), 9(b), 9(c), 9(d), 9(e), 9(f), 9(g), and 9(h) show the trajectory of changes in agricultural cultivation areas and cattle herds within the municipalities hosting the conservation units. This figure reveals an upward trajectory in agricultural lands during the analyzed period. Particular emphasis is warranted for the municipalities of Chapadão do Céu (Figure 9(d)) and Mineiros (Figure 9(g)) in the state of Goiás. In contrast, the livestock data point to a declining trend in cattle herds. Notably, stability is observed solely within the municipalities of Mineiros in Goiás and Alcinópolis (Figure 9(a)) in the state of Mato Grosso do Sul.

These trends reflect a transformation in the landscape, wherein agriculture is on the rise, and cattle populations are decreasing across most municipalities. A distinctive case is the municipality of Costa Rica (Figure 9(f)), located in the state of Mato Grosso do Sul. Here, agricultural expansion over the studied period coincides with a pronounced reduction in cattle numbers starting in 2002.

## 5. Discussion

The effective conservation of native vegetation within the boundaries of NPA underscores their role in safeguarding natural resources, particularly in terms of vegetation preservation, as revealed by remote sensing data. However, the observed impacts in the BZs raise concerns about edge effects on vegetation, with subsequent implications for both the physical and biotic environments due to the effects on habitat diversity. This aligns with findings by Silva et al. [27], who also noted that deforestation within NPA was comparatively lower than in the BZ. Despite covering a substantial portion of the Cerrado region, NPA still fall short of achieving optimal standards, showing uneven distribution, corridor deficiencies, and clustering in specific areas while leaving gaps elsewhere [28].

Observations of study area highlight a resurgence of agricultural activities within NPA boundaries. The presence of such activities could pose a threat to biological diversity and compromise the ecosystem services provided by these areas. This phenomenon was also observed in the studies by Garcia and Ballester et al. [29], Pfaff et al. [30], and Carranza et al. [31] and illustrates that the expansion of agriculture and urbanization remains the primary catalyst for land use changes and consequent natural area loss in the region.

The identification of fire-prone areas within Emas National Park (ENP) in 2018 raises concerns, considering that this is the largest Cerrado conservation unit in Brazil and has historically experienced devastating fires, as indicated by satellite imagery analysis spanning from 1973 to 1995 [32]. Beyond harming native vegetation, fires impact crucial ecosystem processes such as nutrient cycling, climate regulation, and local fauna. These fires can result in reduced species richness and diversity among birds [33], rodents [34], and small mammals [35]. Arthropod responses vary, with some exhibiting positive, negative, or neutral trends [36].

Primarily, the pressures observed in the BZs of can be attributed to agricultural production. Data from the Brazilian Institute of Geography and Statistics (IBGE) indicate that during the study period, agricultural production surged by up to 50%. In contrast, livestock values displayed variability, sometimes experiencing notable declines in certain regions. This dynamic signifies that the expansion of cultivated areas and cattle pastures places pressure on natural habitats via vegetation removal [29, 37–39].

The expansion of cultivated and pasture areas could result in resource depletion, leading to substantial shifts in vegetation composition, alongside adverse impacts on hydrological processes [40]. Such landscape alterations may induce changes in biodiversity and increase the ecological vulnerability of forest fragments. Consequently, the reduction in reserve size could adversely affect the bird community and decrease floral species abundance [41, 42].

Maintaining connectivity between various natural areas is pivotal for gene and species flow, ensuring ecosystem diversity and resilience. However, matrix degradation might impede animal movement and seed dispersal, hindering landscape connectivity and subsequently affecting biodiversity in protected areas [43]. Hakkila et al. [44] emphasize the necessity for conservation policies to consider not only the protected areas themselves but also the surrounding landscape, fostering long-term connectivity and biodiversity preservation.

While the implementation of NPA has yielded positive impacts on landscape dynamics, including increased native vegetation across all protected areas, it is imperative to acknowledge that changes in BZs could threaten NPA due to external pressures. Hence, continuous monitoring and assessment of these regions is crucial. It is worth noting that inadequate resources and unclear management strategies can compromise the effectiveness of NPA, reducing their impact to a symbolic level without achieving their intended objectives.

This scenario underscores the conflicts between economic development and biodiversity conservation, which are evident in the municipalities neighboring NPA and their respective BZs. The proliferation of Pasture and Silviculture areas in these zones illustrates the challenge of protecting these areas, as adherence to regulations does not guarantee their preservation. Existing public policies have been insufficient in safeguarding BZs [45].

Transitions in land use around protected areas often involve conversions to agriculture and livestock, increasing anthropogenic impacts on the function of these areas. In contrast, areas within conservation units are more effective in preserving forest cover. It is demonstrated that increased use of agricultural land within the boundaries of the protected area may be the cause of this conflict and reveals a similar assumption to our study [46].

Protected areas play a crucial role in mitigating vegetation loss, but are insufficient to curb widespread manipulation, as described by Ayach et al. [47]. It is essential to implement public policies that include economic and legal incentives for conservation, in addition to focusing on landscape connectivity to guarantee biodiversity in the long term.

A green infrastructure matrix that integrates both protected areas and their BZs appears as a strategic approach to promoting ecological connectivity [48]. Such a matrix could include corridors of native vegetation, reforestation areas, and sustainable agricultural practices that conserve biodiversity. Improving connectivity allows the free movement of species between habitats, which is essential for maintaining genetic diversity and ecosystem resilience.

Thus, nature conservation is a reality more perceived in the federally owned NPA. This motivates a debate about the fact that local political forces negatively influence natural resource conservation policies. This in turn could weaken state and municipal monitoring of NPA. Despite this, it cannot be denied that NPA play a crucial role in preserving biodiversity and protecting ecosystems. Therefore, protecting and expanding these areas is essential to guarantee a sustainable future for our planet.

## 6. Conclusions

In conclusion, this study provides a foundation for creating spatial databases for deforestation analysis, a crucial tool for monitoring and mitigating deforestation. The establishment of various types of NPA is only the beginning; ongoing management including the development of management plans and area monitoring is essential for ensuring their effectiveness.

Furthermore, the data confirm the hypothesis that can contribute to minimizing the process of landscape transformation in the Cerrado.

## Figures and Tables

**Table 1:** Conservation units studied and their corresponding extents (measured in hectares).

Conservation unit	Area (ha)
Emas National Park	133,069.00
Nascentes do Rio Taquari State Park	30,613.00
Serra do Bom Jardim Municipal Natural Monument	5597.60
Serra do Bom Sucesso Municipal Natural Monument	2665.40
Templo dos Pilares Municipal Natural Park	99.70
Salto do Sucuriú Municipal Natural Park	70.90
Salto da Lage Municipal Natural Park	6.35

*Note:* Source: SISLA/MS.

**Table 2:** Error matrix for 2018 resulting from supervised classification.

Classes	Forest	Savanna	Grassland	Pasture	Temporary crop	Water	Urban infrastructure	Total
Forest	25	0	0	0	2	0	0	27
Savanna	1	20	0	4	3	0	0	28
Grassland	0	0	10	6	1	0	0	17
Pasture	0	0	0	40	8	0	0	48
Temporary crop	0	0	0	7	53	0	0	60
Water	0	0	0	0	0	3	0	3
Urban infrastructure	0	0	0	0	0	0	2	2
Total	26	20	10	57	67	3	2	185

## Data Availability

The data are available at the following links: agriculture: https://sidra.ibge.gov.br/Table/5457; livestock: https://sidra.ibge.gov.br/Table/3939.
